# Acknowledgment of Reviewers

**DOI:** 10.2188/jea.JE20210460

**Published:** 2021-12-05

The following scientists assisted the journal by reviewing manuscripts during the period November 1, 2020 to October 31, 2021.

We gratefully acknowledge their critical evaluation and assistance in selecting articles for publication.

**Table:** 

Abe, Sarah K.
Abe, Takumi
Adachi, Hisashi
Ailes, Elizabeth
Ando, Emiko
Anzai, Tastuhiko
Araki, Atsuko
Asai, Atsushi
Asakura, Keiko
Asayama, Kei
Ayo-Yusuf, Olalekan
Baba, Sachiko
Budhathoki, Sanjeev
Cable, Noriko
Calderon, Berniza
Cho, Sung-il
Choi, Kelvin
Chuang, Shao-Yuan
Cooray, Upul
Cui, Fuqiang
Darrow, Lyndsey
Daube, Mike
DiLorenzo, Madeline
Eguchi, Eri
Eshak, Ehab
Forsum, Elisabet Katarina
Fujii, Ryosuke
Fujita, Yuki
Fujiwara, Aya
Fujiyoshi, Akira
Fukami, Ako
Fukasawa, Maiko
Fukuda, Yoshiharu
Fukui, Keisuke
Fukunaga, Ichiro
Furuta, Michiko
Gao, Wenjing
Geng, Zhi
Gerő, Krisztina
Gibo, Koichiro
Gilmour, Stuart
Glantz, Stan
Guo, Jing
Hachiya, Tsuyoshi
Hamazaki, Kei
Hambidge, K Michael
Hanaoka, Kazumasa
Harada, Kouji
Harville, Emily
Hashimoto, Hiroya
Hata, Jun
Hatakeyama, Toshihiro
Hatekeyama, Shuji
Hattori, Satoshi
Hayashi, Katsuma
Hayashi, Kenichi
Hedman, Linnea
Hermanussen, Michael
Hidaka, Akihisa
Hikichi, Hiroyuki
Hirao, Susumu
Hirata, Takumi
Hirose, Masayo
Hisamatsu, Takashi
Honda, Takanori
Hori, Ai
Hori, Megumi
Horikoshi, Yuho
Hosoda, Masahiro
Hosokawa, Rikuya
Hosozawa, Mariko
Ide, Kazushige
Igeta, Masataka
Ihara, Kazushige
Ihira, Hikaru
Iida, Tadayuki
Iizuka, Ai
Ikeda, Nayu
Imamura, Haruhiko
Inada, Haruhiko
Inoue, Kosuke
Inoue, Taku
Inoue, Yosuke
Ishikawa, Hideki
Ishikawa, Hirofumi
Ishimaru, Miho
Ishimaru, Tomohiro
Ishizaki, Tatsuro
Isumi, Aya
Itani, Osamu
Ito, Hidemi
Ito, Masanori
Ito, Tsubasa
Iwagami, Masao
Iwamoto, Momoko
Iwasa, Hajime
Iwasaki, Masanori
Iwata, Atsushi
Jung, Minyoung
Kabasawa, Keiko
Kabeya, Yusuke
Kakamu, Takeyasu
Kakizaki, Masako
Kanamori, Satoru
Kanatani, Kumiko
Kase, Tetsuo
Kashino, Ikuko
Katagiri, Ryoko
Katanoda, Kota
Kato, Tuguhiko
Kawado, Miyuki
Kawai, Hisashi
Kelly, Scout
Kenzaka, Tsuneaki
Kihara, Tomomi
Kikuchi, Hiroyuki
Kikuchi, Shogo
Kikuya, Masahiro
Kimura, Yasumi
Kitabatake, Yoshinori
Kitano, Naomi
Kobashi, Gen
Kobayashi-Cuya, Kimi
Kojima, Masayo
Korda, Rosemary J
Kubota, Chika
Kubota, Yasuhiko
Kunishima, Hiroyuki
Kurita, Noriaki
Kusama, Taro
Kuwahara, Keisuke
Kyoung Hwa, Ha
Lai, Edward Chia-Cheng
Lugo, Alessandra
Maeda, Toshiki
Malaty, Hoda
Man, Kenneth
Maruyama, Koutatsu
Matsubara, Shigeki
Matsui, Hiroki
Matsumoto, Naomi
Matsuyama, Yusuke
Metoki, Hirohito
Michikawa, Takehiro
Miller, Connor
Minami, Yuko
Mitsuda, Naomi
Miyake, Yoshihiro
Miyamoto, Yoshihiro
Miyamoto, Yoshihisa
Miyatake, Nobuyuki
Miyazaki, Makoto
Morikawa, Nagisa
Morisaki, Naho
Morita, Ayako
Murakami, Keiko
Murakami, Michio
Muraki, Isao
Murata, Kenji
Muto, Go
Nagamine, Yuiko
Nagao, Masanori
Nagata, Chie
Nagata, Chisato
Nakagomi, Atsushi
Nakamizo, Tomoki
Nakamoto, Isuzu
Nakamura, Yosikazu
Nakanishi, Miharu
Nakata, Yoshio
Nakatochi, Masahiro
Nakaya, Naoki
Narisada, Akihiko
Nawa, Nobutoshi
Nemoto, Yuta
Nishino, Yoshikazu
Nishioka, Yuichi
Nishiyama, Takeshi
Noguchi, Taiji
Noma, Hisashi
Norful, Allison
Oba, Mari
Oba, Shino
Ochi, Manami
Oh, Jin-Kyoung
Ohfuji, Satoko
Ohsawa, Masaki
Okada, Yohei
Okamura, Tomonori
Oksanen, Tuula
Okui, Tasuku
Okumura, Yasuyuki
Ono, Sachiko
Orui, Masatsugu
Osorio, Julio
Ostroumova, Evgenia
Otani, Takahiro
Otsuka, Rei
Ozasa, Kotaro
Oze, Isao
Park, Sehoon
Purohit, Bharathi M.
Rasmussen, Svein
Rudolph, Jacqueline
Saigusa, Shin
Saijo, Yasuaki
Sairenchi, Toshimi
Saito, Isao
Saito, Junko
Sakamoto, Haruka
Sakamoto, Naoko
Sakata, Ritsu
Sasai, Hiroyuki
Sasaki, Natsu
Sasaki, Yuri
Sata, Fumihiro
Sato, Nobuhiro
Sato, Shinichiro
Sato, Shuntaro
Sato, Tomoharu
Sato, Yukihiro
Satoh, Michihiro
Schlaud, M
Schwartz, Sharon
Shiba, Koichiro
Shimazaki, Yoshihiro
Shimazu, Taichi
Shimizu, Yuji
Shinozaki, Tomohiro
Shiraishi, Atsushi
Shiraishi, Nao
Shitara, Kohei
Shypailo, Roman J.
Sloan, Robert
Smith, Louisa
Sobue, Tomotaka
Sohn, Chul
Sone, Tomofumi
Stival, Chiara
Subramanian, SV
Sugasawa, Shonosuke
Sugawara, Yumi
Sugiyama, Daisuke
Sugiyama, Hiromi
Sugiyama, Kemmyo
Suzuki, Etsuji
Suzuki, Futoshi
Suzuki, Harumitsu
Suzuki, Kohta
Suzuki, Sadao
Svensson, Thomas
Tachimori, Hisateru
Taguri, Masataka
Takada, Midori
Takahashi, Kunihiko
Takaku, Reo
Takao, Soshi
Takatorige, Toshio
Takeuchi, Kenji
Takimoto, Hidemi
Tamura, Takashi
Tanabe, Naohito
Tanaka, Hirokazu
Tanaka, Junko
Taniguchi, Yu
Tanihara, Shinichi
Tanno, Kozo
Thorburn, David
Tomata, Yasutake
Tran, Binh Thang
Tsuboya, Toru
Tsuji, Taishi
Tsutsumi, Akizumi
Ueda, Peter
Ueda, Yutaka
Uehara, Ritei
Uemura, Hirokazu
Ukawa, Shigekazu
Wada, Ichiro
Wada, Keiko
Watanabe, Daiki
Wu, Wei-Te
Yamada, Masaaki
Yamada, Michiko
Yamagishi, Kazumasa
Yamaguchi, Miwa
Yamakita, Mitsuya
Yamamoto-Hanada, Kiwako
Yamauchi, Takashi
Yatsuya, Hiroshi
Yokomichi, Hiroshi
Yokoyama, Tetsuji
Yokoyama, Yuri
Yoneoka, Daisuke
Yorifuji, Takashi
Yoshida, Daigo
Yoshinaga, Shinji
Yoshioka, Eiji
Zaidi, Jaffer
Zaitsu, Masayoshi
Zha, Ling
Zhan, Siyan
Zhou, Zhonghui

